# Participation in early childhood education and care in Finland mitigates the associations between maternal psychological distress and child social and emotional problems at age two

**DOI:** 10.1007/s00787-025-02865-9

**Published:** 2025-10-15

**Authors:** Katja Tervahartiala, Riikka Korja, Vilma Sarelius, Tuomo-Artturi Autere, Hasse Karlsson, Alice S. Carter, Linnea Karlsson, Saara Nolvi

**Affiliations:** 1 Department of Psychology and Speech-Language Pathology, University of Turku, Turku, Finland; 2 Department of Psychology, University of Jyväskylä, Jyväskylä, Finland; 3 Centre of Excellence in Learning Dynamics and Intervention Research (InterLearn), University of Jyväskylä and University of Turku, Turku, Finland; 4 FinnBrain Birth Cohort Study, Turku Brain and Mind Center, Department of Clinical Medicine, University of Turku and Turku University Hospital, Turku, Finland; 5 Department of Psychiatry, University of Turku and Turku University Hospital, Turku, Finland; 6 Department of Child Psychiatry, University of Turku and Turku University Hospital, Turku, Finland; 7 Centre for Population Health Research, University of Turku and Turku University Hospital, Turku, Finland; 8 Department of Clinical Medicine, Unit of Public Health, University of Turku and Turku University Hospital, Turku, Finland; 9 Department of Psychology, University of Massachusetts, Boston, MA USA

**Keywords:** Social and emotional problems, Socio-emotional competence, Psychological distress, Early Childhood Education and Care (ECEC), Family-based (ECEC)

## Abstract

It is well-established in previous research that maternal psychological distress may have adverse effects on child social and emotional development. However, less is known about the environmental protective factors on child outcomes. This study aimed to explore the moderating role of Early Childhood Education and Care (ECEC) participation on the associations between maternal long-term prenatal, postnatal or current psychological distress and child social and emotional problems and competence. A total of *1*,*191* children (*M*_age_ = 24.54 months, *SD* = 0.59; 47.4% girls) were drawn from the FinnBrain Birth Cohort Study in Finland. Children participated either in center-based or family-based ECEC or they were cared for at home. Mothers had filled in self-reports of their long-term psychological distress during pregnancy, and postpartum, and the evaluation of their child social and emotional problems and competence at the child age of 2 years. The results showed that the ECEC participation played a protective role in the relation between current maternal psychological distress and child social and emotional problems. Associations between maternal symptoms and children’s social and emotional problems was attenuated for children who participated in center-based ECEC (β = -0.37, [-0.64, -0.09], *p* = 0.008), or family-based ECEC (β = -0.75, [-1.11, -0.40], *p* < 0.001) when compared to children who were cared for at home. This study reinforces our understanding of the ECEC’s potential buffering role in at-risk families and in particular in Nordic countries characterized by high-quality of ECEC and high enrollment rates during early childhood.

**Supplementary Information:**

The online version contains supplementary material available at 10.1007/s00787-025-02865-9.

## Introduction

Child social and emotional problems affect between 7% and 25% of children in early childhood, making them an important target for child healthcare [1, 2]. These problems are typically categorized into internalizing and externalizing behaviors, with the latter characterized by symptoms of anxiety, depression and withdrawal, and the former by conduct problems, impulsivity, and disruptive behavior [3, 4]. The emergence of social and emotional problems can lead to difficulties in peer relations, deficits in school performance, and later mental health disorders [5].

Socio-emotional competence, in turn, manifests as an age-appropriate emotion regulation and ability to collaborate with others [5]. Typically, a child with high socio-emotional competence make easily friends, is cooperative with others, and has emotional competence, i.e., ability to understand others feelings and capacity to regulate own positive and negative emotions [6, 7]. Children generally have a need to engage in play and interact with peers. Early childhood settings, such as ECEC, play an important role in enabling this. Development in children is shaped by the continuous interaction between their individual characteristics and environmental contexts [8]. Early emerging socio-emotional competence has been viewed as a protective factor, and social and emotional problems as risk factors for a range of later child outcomes [7]. Because of the high prevalence of social and emotional problems in early childhood, it is important to identify contributors to these problems as well as potential environmental protective factors.

Maternal pre- and postnatal psychological distress, such as depressive and anxiety symptoms, are key correlates of higher levels of child social and emotional problems and lower competence during toddlerhood and preschool age [9–13]. Further, pre- and postnatal psychological distress is hypothesized to influence child development through different mechanisms. Prenatal distress can be transmitted through the programming of fetal HPA axis functioning during pregnancy [14], thus affecting child stress regulation and emotional reactivity, ultimately impacting socio-emotional development [11, 13, 15–17]. Maternal postnatal psychological distress, in turn, may affect child development through parenting practices and inconsistent or challenged parent-child interactions [18–20]. For example, maternal postpartum distress is suggested to have negative effects on a mother-infant bonding, a mother’s attachment style, as well as her sensitivity and attunement towards a child’s needs, which are all crucial in supporting child socio-emotional development [21–23].

Given that 10–40% of mothers experience psychological distress symptoms during pregnancy and in the early postnatal years [24], it is important to investigate how the impact of these symptoms on children can be mitigated. Recently, the focus has shifted to environmental protective and resilience factors for families with young children [25]. Research has shown that cooperation with friends and relatives, as well as social support from the professionals, reduces the effects of maternal distress on children’s social and emotional problems [25–27]. Additionally, our recent review indicated that environmental factors, such as high parental SES and social support, protect children from altered brain development that may compromise emotion regulation, thereby impacting socio-emotional development [28].

The investigation of families’ childcare arrangements is of particular relevance, as most children in industrialized countries participate in non-parental out-of-home childcare [i.e., Early Childhood Education and Care (ECEC)]. Although much emphasis has been placed on the research of possible risks associated with early ECEC [29–32], many studies also indicate that it has positive effects, particularly on a child’s cognitive and language skills [29, 33, 34], as well as academic achievement and school readiness [35, 36]. ECEC is especially beneficial for children who experience challenges in their primary family environments [37].

So far, only a few studies have shown that participation in ECEC protects children from the negative effects of maternal depressive symptoms [26, 38–43]. In particular, high-quality ECEC protected children from clinically relevant maternal depression in early childhood. Children participating higher-quality ECEC exhibited fewer problem behaviors — such as hyperactivity, inattention or externalizing symptoms — compared to their counterparts in lower-quality care. Interestingly, ECEC quality was not associated with child socio-emotional outcomes for children whose mothers did not report any symptoms [38]. ECEC participation may also protect a child from broader environmental adversity, not just maternal psychological distress. Earlier research suggests that children from low-income families experiencing household chaos, such as disorganization, instability, as well as cumulative risk factors benefited from non-parental, out-of-home ECEC participation [37, 44–46]. For those children, greater hours in ECEC were associated with better executive functioning [47], fewer social and emotional problem behavior [37], and more balanced stress regulation [48–50]. Importantly, ECEC participation increased children’s resilience and protected them from the long-term adverse effects of early family risks compared to children who did not participate in ECEC [46]. In contrast, Paquin and colleagues (2020) [51] did not find any buffering effects of ECEC participation on the associations between maternal depressive symptoms and children’s cognitive skills. However, ECEC participation still improved children’s academic achievement and school readiness. Hence, more research is needed to clarify complex relationships between maternal psychological distress (both pre- and postnatal), child’s socio-emotional development, and ECEC participation.

This study was conducted in Finland, where the ECEC enrolment rate is approximately 37–42% for children aged 0–2 years and 88–90% for children aged 3–5 years [52, 53]. In other Nordic countries (e.g., Norway and Denmark), enrolment rates are slightly higher, being 54–59% for 0–2-year-olds and 97% for 3–5-year-olds, due to shorter parental allowance systems [52]. In Finland, children under one year old are typically cared for at home and do not participate in ECEC [54]. However, overall ECEC enrolment rates in the Nordic countries (Denmark, Finland, Iceland, Norway and Sweden) exceed the OECD (The Organisation for Economic Co-operation and Development) average [52]. In the Nordic countries, ECEC policy is closely linked to welfare, social policy, and education. These countries have scored highly in educational outcomes, quality of life, and democracy. This success is often referred to as the “Nordic model”, which forms the basis of ECEC and emphasizes democracy, social equality, child-centeredness, play, learning, and professionalism [55, 56]. In Finland, teacher qualifications, staff-to-child ratios, and the pedagogy in ECEC are highly regulated by the government and local authorities. The Finnish National Agency for Education determines the core curriculum for ECEC in accordance with the Early Childhood Education and Care Act [57].

This study aimed to explore the moderating role of ECEC participation on the associations between maternal long-term prenatal, postnatal and current psychological distress and child social and emotional problems and competence at the age of two years.

The specific goals of the current study were as follows:

1. To investigate whether maternal long-term prenatal (gestational weeks 14, 24, and 34), postnatal (at 3, 6, 12 months), or current (at 2 years) psychological distress, indexed by anxiety (SCL-90) and depressive (EPDS) symptoms, is associated with child social and emotional problems and socio-emotional competence at the age of 2 years.
2. To examine the moderating effect of ECEC (center-based or family-based) participation on the potential associations between maternal prenatal, postnatal, or current psychological distress and child social and emotional problems as well as socio-emotional competence.

We hypothesized that:

1. Maternal prenatal, postnatal, and current psychological distress would be associated with increased social and emotional problems and lower socio-emotional competence in children at the age of 2 years.
2. Association between maternal prenatal, postnatal, or current distress symptoms and children’s social-emotional problems and competence is weaker among children who participate ECEC when compared to those cared for at home.

## Methods

### Participants

A total of *1*,*191* children at the age of 2 years were drawn from the FinnBrain Birth Cohort Study (*N* = 3,*808*) in Finland. The FinnBrain is a longitudinal research project which aims to study the influences of early life stress as well as genetic and environmental factors on child brain development, and health, and behavioral outcomes [58]. Recruitment of the mothers and fathers took place during the first ultrasound visit during gestational week 12 by research nurses in Southwest Finland and the Åland Islands. According to the FinnBrain study inclusion criteria, families with a sufficient knowledge of Finnish or Swedish and with a normal ultrasound screening result were enrolled to the study.

### Procedure

**Fig. 1:**
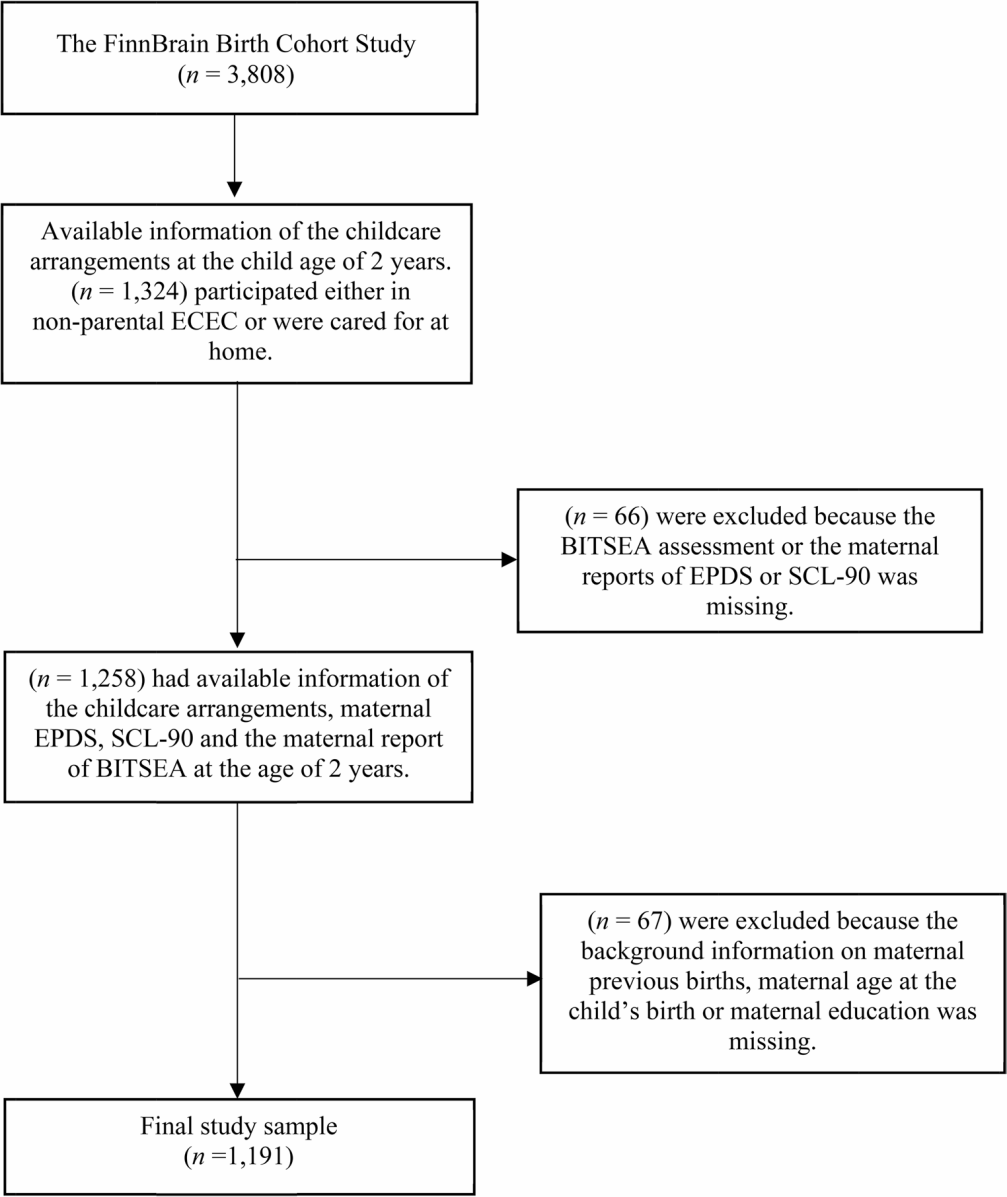
Flowchart of the study sample

The final sample of the current study consisted of children whose mothers had completed the birth cohort research questionnaires, including self-reported psychological distress during pregnancy and postpartum, and the Brief InfantToddler Social and Emotional Assessment (BITSEA) at the child age of 2 years, and who had reported the child’s childcare arrangements (Fig. 1). The children in this study participated in ECEC either in non-parental center-based ECEC (*n* = 508) or non-parental family-based ECEC (*n* = 200), or they were cared for at home (*n* = 483). The center-based ECEC is provided in public or private childcare units, with legislation setting requirements for group sizes and child-to-caregiver ratios. The family-based ECEC is typically organized in the childminder’s own home, caring for a small number of children. Both types of childcare are highly regulated in Finland and are required to follow the national core curriculum for ECEC in accordance with the Early Childhood Education and Care Act [57]. In this study, children who were cared for at home had not yet participated in out-of-home childcare. The primary caregiver at home was most commonly the mother (82.4%), followed by the father (6.8%), a grandparent or another familiar caregiver (7.7%), or the information was missing (3.1%).

All study participants provided written informed consent, and parents gave consent on behalf of their child. The participating families were informed about the study protocol and had the right to withdraw from the study at any time. Each participant of the FinnBrain study was assigned a unique identification code, and all data were processed by using these codes to ensure confidentiality. The findings have been reported in such a way that individual participants cannot be identified. The Ethics Committee of the Hospital District of Southwest Finland has approved the FinnBrain Birth Cohort Study with the protocol number “ETMK: 137/1801/2013.” This study also meets the ethical guidelines and has been performed in accordance with the ethical standards laid down in the 1964 Declaration of Helsinki and its later amendments.

### Measures

#### Brief Infant-Toddler Social and Emotional Assessment

The child’s social and emotional problems and competence were evaluated at the age of 2 years using a maternal report of the Brief InfantToddler Social and Emotional Assessment (BITSEA). The BITSEA questionnaire consists of 42 items designed to screen for developmental problems and assess the socio-emotional competence of children aged 12 to 36 months [4]. The questionnaire includes dimensions measuring internalizing and externalizing symptoms, dysregulation problems, maladaptive behaviors and socio-emotional competence. For this study, only the Total Problem Scale (a summary of problem scales) and the Competence Scale were used. The internal consistency of the selected factors was consistent with previous studies [1, 59] (Total Problem Scale: *α* = 0.70; Competence Scale: *α* = 0.59).

#### Edinburgh Postnatal Depression Scale

Maternal pre- and postnatal depressive symptoms were measured using the Edinburgh Postnatal Depression Scale (EPDS) [60] during pregnancy at gestational weeks (GW) 14, 24 and 34, as well as postnatally when the child was 3, 6, and12 months old. Current symptoms were reported when the child was 2 years.

#### Symptom Checklist-90

Anxiety symptoms were measured using the Symptom Checklist-90 (SCL-90) [61] at GW 14, 24 and 34, and postnatally at the child’s age of 3 and 6 months, with current symptoms reported at 2 years.

The internal consistency of both EPDS and SCL-90 was high, ranging from α = 0.80 to 0.85 across different time points.

#### Background information

Maternal background data were obtained from the self-reported questionnaires collected throughout the FinnBrain Birth Cohort study and from The wellbeing services county of Southwest Finland VARHA registers (www.varha.fi/en). Background information included maternal education, maternal age at the child’s birth, previous childbirths, and the child’s official sex assigned at birth (1 = boy, 2 = girl).

### Data analysis

Bivariate analyses were conducted using Spearman correlation, independent samples t-tests, and one-way ANOVA to identify which background variables were associated with children’s scores on the BITSEA Total problem and Competence scales. Maternal level of education, child’s sex, maternal age at child’s birth, and number of previous births were chosen as covariates based on these preliminary analyses. Maternal EPDS and SCL-90 scores were collected at three different time periods: prenatal (GW 14, 24, 34), postnatal (3, 6, 12 months), and 2 years of age. EPDS and SCL-90 scores were correlated for prenatal measurements between *r* = 0.47 − 0.69, for postnatal measurements between *r* = 0.52 − 0.68, and for 2 years of age *r* = 0.65. For each time period, a combined measure of maternal psychological distress symptoms was formed by summing standardized EPDS and SCL-90 scores. This was done by first standardizing the individual scores and then adding the standardized scores together within each period. Thus, three measures - prenatal maternal distress symptoms, postnatal maternal distress symptoms, and 2-year maternal distress symptoms - were formed and used in the models. The mean scores of EPDS and SCL-90 values at different measurement points are presented in the Supplement 2.

General linear models (GLM) were used to examine the associations between the two BITSEA scales (Total problem scale and Competence scale) and maternal pre-, and postnatal, and current psychological distress symptoms as well as ECEC settings (center-based ECEC, or family-based ECEC or home care setting). The models were created for each period of maternal symptoms (pre-, and postnatal- and current), and BITSEA scales, resulting in a total of six models (Study question 1). All the chosen covariates were included in the models.

**Fig. 2:**
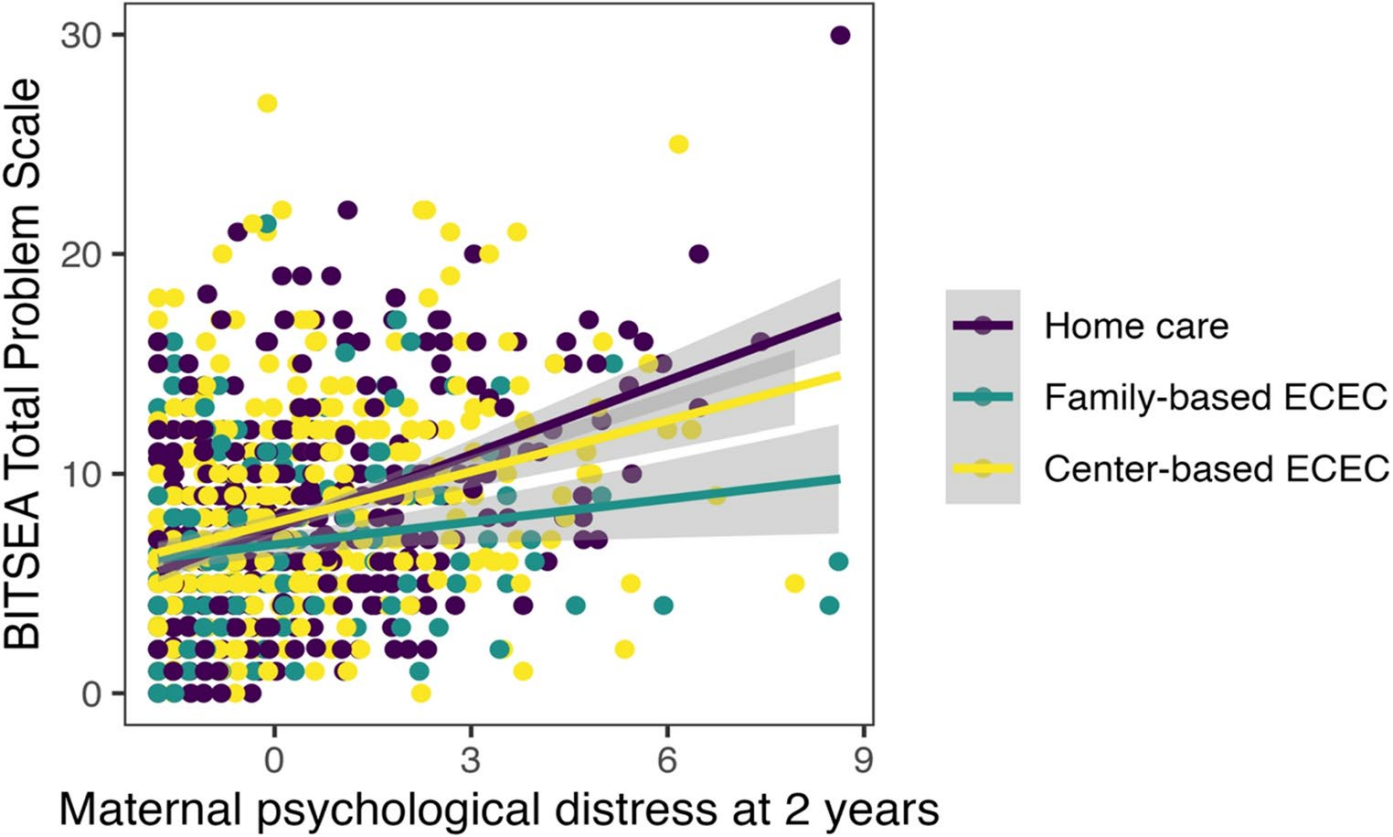
Associations between maternal current psychological distress and BITSEA Total problem scale and ECEC settings

Next, the interaction term between ECEC settings and maternal psychological distress symptoms were included in these models, resulting in six additional models to test the moderating effect of ECEC setting on the association between maternal psychological distress and child socio-emotional outcomes (Study question 2). Given the skewed distribution of maternal psychological distress symptoms, bias-corrected and accelerated (BCa) bootstrap confidence intervals [62], based on 5,000 bootstrap samples, were calculated for the regression parameters. BCa confidence intervals were chosen because they do not rely on the assumption of normality in the residuals. The same background variables were controlled for in all models, which are detailed in Table 2. Preliminary analyses were done with SPSS version 27 [63], while the linear models were analyzed with R version 4.2 [64]. Bootstrap confidence intervals were calculated using the boot package [65, 66]. The ggplot2 package [67] was used to produce Fig. 2.

## Results

All the participants were Finnish, and both maternal origin and native language were predominantly Finnish. No significant differences were found between the ECEC groups in the child’s age, child’s sex, maternal pre-, postnatal or current psychological distress symptoms, maternal age at the child’s birth, or scores on the BITSEA competence scale. However, group differences were observed in the child’s age at ECEC entry, scores on the BITSEA total problem scale, the number of mothers’ previous births, and the maternal level of education (Table 1).

**Table 1:** Demographic characteristics of the participants

	Center-based ECEC	Family-based ECEC	Home care	Total sample	*P*-value
Sample *N*	508	200	483	1191	
Child age (months), mean (SD)	24.58 (0.67)	24.51 (0.43)	24.51 (0.56)	24.54 (0.59)	0.103
Child sex (girls), *N* (%)	241 (47.4%)	92 (46.0%)	219 (45.3%)	552 (46.3%)	0.798
Age (months) at ECEC entry, mean (SD) [interquartile range]	17.26 (4.20) [14–21]^1^	15.35 (4.10) [12–18]^2^		16.27 (4.25) [13–20]	< 0.001
BITSEA Total Problem Scale, mean (SD)	7.72 (4.32)	6.76 (3.76)	7.46 (4.40)	7.46 (4.27)	0.027
BITSEA Competence Scale, mean (SD)	18.10 (2.32)	18.37 (2.41)	17.99 (2.56)	18.10 (2.43)	0.176
*Maternal characteristics*					
Maternal age at child’s birth, mean (SD)	31.30 (3.99)	30.92 (4.23)	30.94 (4.68)	31.09 (4.33)	0.332
Number of previous births, mean [range]	0.59 [0–3]	0.49 [0–2]	0.68 [0–7]	0.61 [0–7]	0.014
Education *N* (%)					
High school/Vocational education	101 (19.9%)	58 (29.0%)	184 (38.1%)	343 (28.8%)	< 0.001
Applied university	147 (28.9%)	50 (25.0%)	148 (30.6%)	345 (29.0%)	
University degree	260 (51.2%)	92 (46.0%)	151 (31.3%)	503 (42.2%)	

^1^n = 506

^2^n = 199

### Associations between maternal psychological distress and child social and emotional problems and competence

Maternal pre- (β = 0.28, 95% CI [0.23, 0.33], *p* < 0.001), and postnatal (β = 0.35, 95% CI [0.29, 0.42], *p* < 0 0.001), and current psychological distress (β = 0.81, 95% CI [0.69, 0.94], *p* < 0.001) were associated with child’s higher BITSEA Total Problem Scale at 2 years. Similarly, maternal pre- (β = −0.05, 95% CI [−0.08, −0.02], *p* = 0.002), and postnatal (β = −0.10, 95% CI [−0.14, −0.06], *p* < 0.001), and current psychological distress (β = −0.15, 95% CI [−0.23, −0.08], *p* < 0.001) were associated with child’s lower BITSEA Competence Scale at the same age.

### Moderating role of ECEC participation

Moderation analysis showed that participation in ECEC significantly moderated the associations between maternal psychological distress at 2 years and the concurrent child’s social and emotional problems for family-based ECEC: (β = −0.75, 95% CI [−1.11, −0.40], BCI [−1.14, −0.36], *p* < 0.001), and for center-based ECEC: (β = −0.37, 95% CI [−0.64, −0.09], BCI [−0.70, −0.05], *p* = 0.008) (Table 2). In other words, association between maternal current psychological distress and children’s social and emotional problems was attenuated for children who participated either family-based or center-based ECEC when compared to their counterparts who were cared for at home (Fig. 2). The standardized coefficient (effect sizes) were − 0.32 for family-based ECEC and − 0.16 for center-based ECEC, reflecting moderate and small effect sizes.

**Table 2:** Linear models with interaction term between maternal psychological distress and BITSEA total problem scale and ECEC settings

Total Problem Scale (BITSEA)	Maternal prenatal distress (gw. 14, 24, 34)			Maternal postnatal distress (3, 6, 12 months)			Maternal current distress (2years)			
	β	95% CI	*P*	β	95% CI	*P*	β	95% CI	95% BCI	*P*
Intercept	8.70	6.89 − 10.51	< 0.001	9.70	7.81−11.59	< 0.001	9.65	7.92 − 11.38	7.79–11.31	< 0.001
Child’s sex (girl vs. boy)	−0.64	−1.11− −0.17	0.008	−0.86	−1.35 − −0.37	0.001	−0.59	−1.04 − −0.14	−1.03 – −0.15	0.010
Maternal age at birth	−0.01	−0.07–0.05	0.737	−0.05	−0.11–0.01	0.130	−0.04	−0.10–0.02	−0.10–0.02	0.164
Maternal education										
Mid vs. low	−0.98	−1.62 – −0.34	0.003	−0.73	−1.40 – −0.06	0.034	−0.79	−1.40 – −0.17	−1.42–0.16	0.012
High vs. low	−0.82	−1.44 – −0.20	0.009	−0.40	−1.05–0.25	0.231	−0.58	−1.17–0.01	−1.17–0.02	0.056
Mother’s previous births	−0.33	−0.65 – −0.02	0.037	−0.11	−0.42–0.21	0.513	−0.25	−0.54–0.05	−0.53–0.04	0.109
Maternal prenatal distress (gw. 14, 24, 34)	0.29	0.21–0.36	< 0.001							
Maternal postnatal distress (3, 6, 12 mo)				0.37	0.27–0.46	< 0.001				
Maternal current distress (2years)							0.81	0.69–0.94	0.90–1.34	< 0.001
Family-based ECEC vs. home care	−0.59	−1.28–0.10	0.096	−0.84	−1.57 – −0.12	0.022	−0.79	−1.40 – −0.17	−1.38 – −0.13	0.012
Center-based ECEC vs. home care	0.69	0.16–1.22	0.010	0.33	−0.22–0.88	0.243	−0.58	−1.17–0.01	−0.24–0.81	0.056
Family-based ECEC x maternal prenatal distress (vs. home care)	−0.10	−0.24–0.04	0.176							
Center-based ECEC x maternal prenatal distress (vs. home care)	0.02	−0.09–0.12	0.780							
Family-based ECEC x maternal postnatal distress (vs. home care)				−0.08	−0.27–0.11	0.40				
Center-based ECEC x maternal postnatal distress (vs. home care)				0.00	−0.13–0.13	0.994				
Family-based ECEC x current distress at 2y (vs. home care)							−0.75	−1.1 – −0.40	−1.14 – −0.36	**< 0.001**
Center-based ECEC x current distress at 2y (vs. home care)							−0.37	−0.64 – −0.09	−0.70 – −0.05	**0.008**

β = Unstandardized regression coefficient

95% CI = 95% Confidence Intervals

95% BCI = 95% Bootstrap Confidence Intervals

P = Statistical significance at *p* < 0.05

However, participation in ECEC did not moderate the associations between maternal pre- or postnatal psychological distress and child social and emotional problems (Table 2). Additionally, no moderation effects were found for pre-, postnatal or current distress and child socio-emotional competence (see Supplement 1).

## Discussion

The main aim of this study was to investigate the protective role of Early Childhood Education and Care (ECEC) participation for 2-year-old children exposed to maternal pre-, postnatal, or current psychological distress. In line with our expectations, this study showed that maternal pre-, postnatal, and current psychological distress was associated with increased mother-reported child social and emotional problems and lower socio-emotional competence. These results confirm the ample previous evidence on the associations between maternal psychological distress and child outcomes [11–13]. Our results further suggest that participation in ECEC protects children in cases where their mothers experience current psychological distress. Specifically, association between maternal distress symptoms at the child age of 2 years and child social and emotional problems was mitigated in children who participated in ECEC. This finding has important implications for understanding the role of ECEC in at-risk families, particularly in Nordic countries, where high-quality of ECEC and high enrollment rates during early childhood are prominent features.

Interestingly, our study found that ECEC participation protected children only from current maternal distress, but not from past or prenatal stress. It appears that ECEC protects children from maternal distress when symptoms are actively present in the child’s daily life. Time spent in ECEC reduces the time spent at home only and provides additional opportunities for children to form secure relationships with adults outside family context and engage in group activities. This result aligns with earlier research showing similar buffering effects of ECEC in cases of child exposure to maternal psychological distress [26, 38, 40–42]. In particular, high-quality formal and institutional ECEC is suggested to play a crucial protective role in child development [26]. This protective effect can be explained by the ECEC-related features. For example, in higher-quality ECEC settings, teachers have strong skills in supporting children’s play and peer interactions, which helps prevent social and emotional problems in group settings [38]. Earlier findings further indicate that for children facing multiple risk factors in their home environment, ECEC participation is associated with decreased cortisol levels and improved stress regulation capacity [49, 50, 68]. ECEC can provide a secure and predictable setting, including daily routines, as well as respite and necessary support under adverse parenting conditions.

Additionally, a child’s participation in ECEC can benefit parents when their child has social and emotional problems. The maternal protection model was tested in a study of [42] where the girls’ externalizing behaviors at the age of 3.5 years were associated with higher maternal depressive symptoms, and the effect was more pronounced when the child did not participate in ECEC [42]. However, this association was observed only in girls, which may suggest that externalizing behavior in girls is less socially acceptable, leading to greater parental stress and feelings of parental incompetence [42]. Nevertheless, young children’s self-regulation is closely linked with sensitive interactions with their caregivers. Earliers research has shown that maternal distress may lead to higher negative emotionality and lower self-regulation skills in children [11, 16]. Toddlers often exhibit particularly challenging behaviors as a part of normative development, making it difficult for distressed parents to respond appropriately and prevent their stress from affecting their parenting styles [19, 20, 23]. Furthermore, children exposed to maternal distress may have a greater need for supportive care to achieve optimal developmental outcomes. Therefore, the early childhood environment plays even more critical role in mitigating the earlier adverse effects. Negative developmental trajectories can be altered if a child experiences sensitive and supportive caregiving as well as secure attachment during early childhood years [11].Thus, the findings related to current maternal distress may be explained by multiple mechanisms through which formal ECEC benefits both the child and the parent, especially if parents are undergoing strain.

In this study, the mitigated effect of ECEC participation was observed in both family-based and center-based settings, but the effect size was higher in family-based ECEC compared to center-based ECEC. This finding is plausible, as group sizes in family-based ECEC tend to be smaller, allowing especially the youngest children to receive more individualized care, attention and support from caregivers. In contrast, center-based ECEC includes a larger peer group, potentially reducing the level of individual interaction between the child and caregivers. Additionally, some parents may consider family-based ECEC as a more suitable option for younger children, potentially also reducing their own concerns about the child’s development. In Finland, family-based ECEC is a relatively common choice for the youngest children who are just starting in non-parental out-of-home care. However, despites its benefits, the volume of the family-based ECEC has decreased considerably in recent years [53].

Finally, we did not find a moderating effect of ECEC participation on a child’s socio-emotional competence. This is consistent with earlier studies that show very few direct links between ECEC participation and social competence in toddlers [36, 69]. The association between ECEC participation and socio-emotional development is more frequently observed in the problem scale rather than in the competence scale [31]. The lack of findings in the current study may also be explained by the children’s young age, and their limited participation time in ECEC, which may not have been sufficient for it to influence their socio-emotional competence. Future research should follow these children over a longer period to determine whether ECEC has a more pronounced impact on socio-emotional competence as children grow older.

### Limitations

This study has many strengths, such as large sample size and longitudinal measures of maternal psychological distress symptoms. We were also able to research rather young children in three different ECEC settings. However, there are also limitations that should be considered when interpreting the results. First, the effect size concerning the center-based ECEC was small. Furthermore, we only had maternal reports of both her depressive symptoms and child’s behavior, which can result in reporter biases. There may also be additional biases in the child’s behavioral evaluation by mothers with elevated depressive symptom levels. Therefore, it would have been important to receive reports also from the other parent and childcare teachers [70]. We are not aware of whether children showed social and emotional problems also in ECEC or solely at home. Second, although in line with previous studies using the BITSEA measure [1], the Cronbach’s alpha for the BITSEA Competence scale was only moderate (0.59). Third, the children were rather young and only recently started in ECEC, which limit the reliability and generalizability of the findings. It would be important to have longitudinal research in the same participants to examine whether the ECEC’s protective role emerges also for older children or just for toddler who need a lot of support from caregivers. Fourth, we did not have qualitative measures from ECEC units or the children’s home environments. It is well established that in particularly high quality of ECEC has a protective role for children [38]. However, the ECEC quality is considered to be rather high in Finland [71], but surely there are some differences between different ECEC units. Therefore, the generalizability of the results to ECEC units or countries beyond the study context should considered carefully. We had neither data from mother-child interaction or quality of caregiving. This kind of observation could have shed light of the maternal emotional availability and her ability to respond the child’s needs.

## Conclusions

This study found that ECEC participation protected children from the current maternal psychological distress when the children were 2 years old. Association between mother’s depressive and anxiety symptoms and child’s social and emotional problems was weaker in children who participated ECEC. Our study clarifies the role of ECEC in early childhood development, increasing the understanding of the possible protective role of ECEC in case of current distress versus prior stress exposures. In particular, well-organized and high-quality ECEC, along with teachers’ pedagogical competence, may play a crucial role for children from at-risk families. This finding is particularly relevant at a societal level, underscoring the importance of comprehensive support for families with young children.

## Supplementary Information

Below is the link to the electronic supplementary material.

Supplementary Material 1

Supplementary Material 2

## Data Availability

The datasets to reproduce the analyses are not publicly available because of restriction imposed by the Finnish law, and the study's ethical permissions do not allow sharing of the data used in this study. Requests to access the datasets should be directed to the Principal Investigator of the FinnBrain Birth Cohort Study..
