# Supplementary material for: Participation in early childhood education and care in Finland mitigates the associations between maternal psychological distress and child social and emotional problems at age two
Source: Eur Child Adolesc Psychiatry. 2025 Oct 15;35(2):611–21. doi: 10.1007/s00787-025-02865-9 (PMC12957043; doi:10.1007/s00787-025-02865-9)
Supplement: Supplementary file 1 — Supplementary Material 1 [file 787_2025_2865_MOESM1_ESM.docx]

**Supplement 2.**

Mean scores of EPDS and SCL-90 values at different measurement points

|  | **Center-based ECEC** | **Family-based ECEC** | **Home care** | **Total** |
| --- | --- | --- | --- | --- |
| **EPDS, mean (SD)** |  |  |  |  |
| GW 14 | 4.78 (3.81) | 4.56 (3.97) | 5.00 (3.99) | 4.83 (3.91) |
| GW 24 | 4.51 (3.94) | 4.56 (4.00) | 4.79 (3.96) | 4.63 (3.96) |
| GW 34 | 4.33 (3.70) | 4.64 (4.06) | 4.89 (4.34) | 4.61 (4.04) |
| 3 months | 4.24 (3.86) | 4.29 (3.63) | 4.20 (3.82) | 4.23 (3.80) |
| 6 months | 4.39 (4.05) | 4.10 (3.99) | 4.35 (4.38) | 4.32 (4.17) |
| 12 months | 4.95 (4.23) | 4.49 (3.95) | 4.82 (4.11) | 4.82 (4.13) |
| 2 years | 4.56 (4.29) | 4.55 (4.32) | 4.64 (4.32) | 4.59 (4.30) |
| **SCL-90, mean (SD)** |  |  |  |  |
| GW 14 | 2.87 (3.43) | 3.28 (4.37) | 3.25 (4.03) | 3.09 (3.85) |
| GW 24 | 3.55 (3.90) | 3.82 (4.32) | 3.76 (4.30) | 3.68 (4.14) |
| GW 34 | 2.70 (3.46) | 3.24 (3.70) | 3.35 (4.38) | 3.06 (3.91) |
| 3 months | 2.37 (3.16) | 2.47 (2.82) | 2.7 (3.70) | 2.52 (3.35) |
| 6 months | 2.49 (3.62) | 2.67 (3.56) | 2.73 (4.10) | 2.62 (3.81) |
| 2 years | 2.92 (3.86) | 2.89 (4.50) | 2.75 (3.91) | 2.85 (3.99) |
