# Supplementary material for: Participation in early childhood education and care in Finland mitigates the associations between maternal psychological distress and child social and emotional problems at age two
Source: Eur Child Adolesc Psychiatry. 2025 Oct 15;35(2):611–21. doi: 10.1007/s00787-025-02865-9 (PMC12957043; doi:10.1007/s00787-025-02865-9)
Supplement: Supplementary file 2 — Supplementary Material 2 [file 787_2025_2865_MOESM2_ESM.docx]

**Supplement 1.**

| **Competence Scale**  **(BITSEA)** | **Maternal Prenatal distress**  **(gw 14, 24, 34)** | | | **Maternal Postnatal distress**  **(3, 6, 12 months)** | | | **Maternal current distress (2years)** | | |
| --- | --- | --- | --- | --- | --- | --- | --- | --- | --- |
|  | **β** | **95% CI** | ***P*** | **β** | **95% CI** | ***P*** | **β** | **95% CI** | ***P*** |
| Intercept | 19.06 | 17.99 − 20.14 | <0.001 | 18.99 | 17.83−20.16 | <0.001 | 18.86 | 17.83 − 19.90 | <0.001 |
| Child’s sex (girl vs. boy) | 0.85 | 0.57− 1.13 | <0.00 | 0.87 | 0.57 − 1.18 | <0.001 | 0.78 | 0.51 − 1.05 | <0.001 |
| Maternal age at birth | -0.06 | -0.10 – -0.03 | 0.001 | -0.06 | -0.10 – -0.02 | 0.002 | -0.06 | -0.09 – -0.02 | 0.002 |
| Maternal education |  |  |  |  |  |  |  |  |  |
| Mid vs. low | 0.61 | 0.23 – 0.99 | 0.002 | 0.53 | 0.12 – 0.95 | 0.012 | 0.59 | 0.23 – 0.96 | 0.002 |
| High vs. low | 0.65 | 0.28 – 1.01 | 0.001 | 0.60 | 0.20 – 1.00 | 0.003 | 0.63 | 0.27 – 0.98 | 0.001 |
| Mother’s previous births | 0.18 | -0.00 – 0.37 | 0.052 | 0.14 | -0.05 – 0.34 | 0.158 | 0.17 | -0.01 – 0.34 | 0.071 |
| Maternal prenatal distress (gw. 14, 24, 34) | -0.07 | -0.11 – -0.02 | 0.002 |  |  |  |  |  |  |
| Maternal postnatal distress (3, 6, 12 months) |  |  |  | -0.13 | -0.18 – -0.07 | <0.001 |  |  |  |
| Maternal current distress (2years) |  |  |  |  |  |  | -0.21 | -0.32 – -0.09 | 0.001 |
| Family-based ECEC  vs. home care | 0.38 | -0.03 – 0.79 | 0.070 | 0.43 | -0.01 – 0.88 | 0.058 | 0.36 | -0.04 – 0.75 | 0.076 |
| Center-based ECEC  vs. home care | -0.04 | -0.35 – 0.28 | 0.820 | 0.07 | -0.26 – 0.41 | 0.66 | 0.03 | -0.28 – 0.33 | 0.865 |
| Family-based ECEC x maternal prenatal distress (vs. home care) | 0.06 | -0.03 – 0.14 | 0.194 |  |  |  |  |  |  |
| Center-based ECEC x maternal prenatal distress (vs. home care) | 0.03 | -0.03 – 0.10 | 0.330 |  |  |  |  |  |  |
| Family-based ECEC x maternal postnatal distress (vs. home care) |  |  |  | 0.08 | -0.04 – 0.19 | 0.199 |  |  |  |
| Center-based ECEC x maternal postnatal distress (vs. home care) |  |  |  | 0.04 | -0.04 – 0.12 | 0.313 |  |  |  |
| Family-based ECEC x current distress at 2y  (vs. home care) |  |  |  |  |  |  | 0.11 | -0.10 – 0.33 | 0.302 |
| Center-based ECEC x current distress at 2y  (vs. home care) |  |  |  |  |  |  | 0.08 | -0.09 – 0.24 | 0.355 |

Linear models with interaction term between maternal psychological distress and BITSEA Competence Scale and ECEC settings.

β = Unstandardized regression coefficient

95% CI = 95% Confidence Intervals

P = Statistical significance at p < 0.05
